# Structure–Activity Relationships of *N*-Acyl Dopamines in Inhibiting Myofibroblast Transdifferentiation of Retinal Pigment Epithelial Cells

**DOI:** 10.3390/biom15111526

**Published:** 2025-10-30

**Authors:** Dandan Zhao, Vishaka Motheramgari, Riley Freudenberger, Sarah H. Shrader, Lucy J. Sloan, Zoe Lung, Wei Wang, Shigeo Tamiya, Zhao-Hui Song

**Affiliations:** 1 Department of Pharmacology and Toxicology, University of Louisville School of Medicine, Louisville, KY 40292, USA; 2 Department of Ophthalmology and Visual Sciences, University of Louisville School of Medicine, Louisville, KY 40292, USA; 3 Eye Hospital of China Academy of Chinese Medical Sciences, Beijing 100040, China; 4 Department of Ophthalmology and Visual Sciences, Ohio State University College of Medicine, Columbus, OH 43210, USA

**Keywords:** *N*-acyl dopamines, endocannabinoids, myofibroblast transdifferentiation, proliferative vitreoretinopathy (PVR)

## Abstract

Aberrant wound healing in the retina can manifest as proliferative vitreoretinopathy (PVR), which involves the myofibroblast transdifferentiation of retinal pigment epithelial (RPE) cells. In this study, experiments were conducted to examine the structure–activity relationships of endocannabinoid-like compounds, *N*-acyl dopamines, on the myofibroblast transdifferentiation of RPE cells. The collagen matrix contraction assay was used to assess myofibroblast function. Western blot analysis and immunocytochemistry techniques were used to evaluate myofibroblast markers. *N*-palmitoyl dopamine (PALDA), *N*-oleoyl dopamine (OLDA), and *N*-arachidonoyl dopamine (NADA), in a concentration-dependent manner, inhibited contraction of collagen matrices mediated by either primary porcine RPE cells treated with TGF-β2, or human RPE cells treated with TGF-β2 plus TNFα (TNT). The rank order of potency was PLDA = OLDA > NADA. In contrast, the substitution of dopamine with other polar head groups led to a complete loss of their ability to inhibit myofibroblast transdifferentiation. Western blot analysis demonstrated that PALDA, OLDA, and NADA down-regulated the myofibroblast markers fibronectin and α-SMA. Immunocytochemistry experiments showed that these *N*-acyl dopamines reduced the incorporation of α-SMA into F-actin stress fibers. Overall, these structure–activity relationship studies demonstrate that the dopamine head group is crucial for *N*-acyl dopamine to inhibit myofibroblast transdifferentiation of RPE cell, whereas the fatty acid side chain determines the potency of it. This study points to the potential of *N*-acyl dopamines as a novel class of therapeutic agents for treating retinal fibrotic conditions, such as PVR.

## 1. Introduction

Wound healing is an important and complex process that requires the coordinated efforts of many different cell types. This process can become dysregulated in various pathological conditions and lead to the formation of fibrotic tissues [1,2]. In the eye, retinal fibrosis can manifest as proliferative vitreoretinopathy (PVR) [3,4,5].

PVR frequently arises as a complication of rhegmatogenous retinal detachment and is a significant concern following retinal reattachment surgery (postoperative PVR). It is a process of dysregulated wound healing and scarring, characterized by the growth of contractile fibro-cellular membranes within the vitreous cavity and on the surface of the retina [3,4,5].

Despite its severe impact on visual acuity, including cases that can lead to blindness, there are currently no approved medications for the treatment or prevention of PVR. This highlights the urgent need to investigate the underlying pathological molecular mechanisms and to identify potential therapeutics for PVR [3,4,5].

Retinal pigment epithelial (RPE) cells are considered to play a central role in the pathogenesis of PVR. Upon exposure to fibrotic growth factors and cytokines such as TGF-β and TNF-α, RPE cells undergo an epithelial-to-mesenchymal transition and transdifferentiate into myofibroblasts. This process is marked by the incorporation of alpha-smooth muscle actin (α-SMA) into F-actin stress fibers, which enhances cellular contractility [6,7]. The resulting myofibroblasts contribute to extracellular matrix accumulation and contraction, ultimately driving fibrotic membrane formation.

The endocannabinoid system is composed of cannabinoid receptors, endogenous cannabinoids such as anandamide (*N*-arachidonoyl ethanolamide) and 2-arachidonoyl glycerol (2-AG), and the enzymes responsible for their synthesis and degradation [8,9,10].

**Figure 1:**
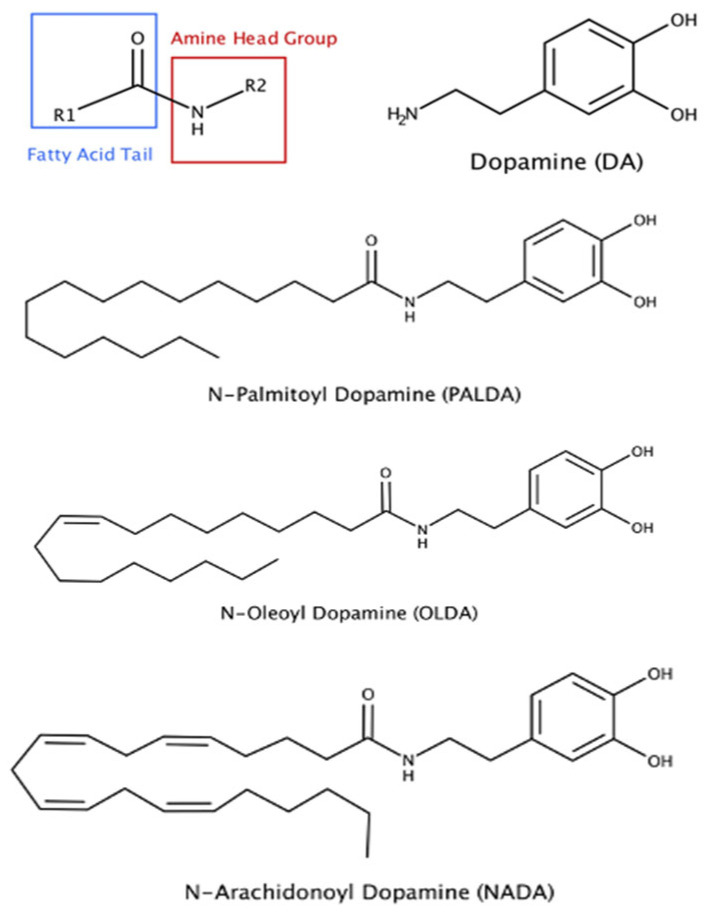
Chemical structures of three *N*-acyl dopamines that are effective in inhibiting myofibroblast transdifferentiation of RPE cells.

The endocannabinoid compounds typically consist of a long-chain fatty acid that can be saturated, like palmitic acid, or unsaturated, like arachidonic acid, that is conjugated to a polar head group (Figure 1). Moreover, a newer class of endocannabinoids have been reported, in which a fatty acid is conjugated via an amide bond to an amino acid (*N*-acyl amino acid) or a neurotransmitter, such as dopamine (*N*-acyl dopamine) [11,12,13].

In our previous work, we discovered that *N*-oleoyl dopamine (OLDA), one of the *N*-acyl dopamines, inhibited TGF-β2-induced myofibroblast transdifferentiation of porcine RPE cells [14]. This previous study suggests that OLDA, and related endocannabinoid-like compounds may have the potential to be developed into new treatments for retinal fibrosis. In this study, experiments are conducted on both porcine and human RPE cells to answer two questions: (1) whether the ability of *N*-acyl dopamine derivatives to inhibit RPE cells transdifferentiating into myofibroblasts may be influenced by the structural characteristics of their fatty acid side chains; and (2) whether the modification of the polar head group may lead to the discovery of other *N*-acyl amide derivatives that are capable of inhibiting transdifferentiation of RPE cells into myofibroblasts.

## 2. Materials and Methods

### 2.1. Porcine and Human RPE Cells

Primary cultures of porcine RPE cells were established from freshly obtained porcine eyes, kindly donated by the Swift Meat Packing Co. (Louisville, KY, USA). The tissue collection conformed to the ARVO statement of the Use of Animals in Ophthalmic and Vision Research. Porcine RPE cells were isolated based on our previously reported methods [14,15]. Briefly, posterior eyecups, prepared from sterilized eyes by removal of the anterior segment, were incubated in 0.5% dispase/5% FBS DMEM/PBS solution for 1 h at 37 °C. RPE sheets flushed out from incubated eyecups were collected and rinsed. These sheets were broken down to small cell clusters by repeated pipetting, and cultured in a Matrigel coated flask, in Dulbecco’s modified Eagle’s medium (DMEM; VWR; Radnor, PA, USA), supplemented with 10% FBS (Sigma-Aldrich, St. Louis, MO, USA), 100 IU/mL penicillin, 100 μg/mL streptomycin, and 10 μM of ROCK inhibitor Y27632 (HelloBio, Princeton, NJ, USA). Adult human primary RPE cells were procured from the Eye-Bank for Sight Restoration (New York, NY, USA) and cultured according to manufacturer’s instructions. RPE cells were routinely passaged using trypsin and were used for contraction assays, Western blot analysis, and immunocytochemical staining at passages 3 or 4 for both porcine and human RPE cells.

### 2.2. Contraction Assays

Cultured RPE cells (passage 3 or 4) were plated on 0.4 mL of solidified neutralized type I collagen (Cellmatrix Type I-A, Fujifilm Wako Chemicals, Richmond, VA, USA) in each well of the 24-well plates. RPE cells in 2.5% fetal bovine serum and DMEM were added to each well at 1 × 10^5^ cells per well and incubated for 4 h to allow for cell attachment. Cells were then pretreated with various *N*-acyl amide derivatives for 15 min and subsequently treated with TGF-β2 (10 ng/mL) for porcine RPE cells, or a combined treatment of TGF-β2 + TNF-α (TNT; 10 ng/mL of each) for human RPE cells. Following 72 h of incubation, collagen gels were gently released from the wells and photographed after 4 h. Images were assessed for gel contraction and quantified using NIH ImageJ Software (version 1.54p). Results are mean ± SEM from 3 to 4 different experiments with different batches of cells.

### 2.3. Western Blot Analysis

Collagen gels with attached RPE cells from the contraction assays were prepared for Western blot analysis using the lysis buffer (20 mM Tris-HCl, pH 7.5, 150 mM NaCl, 1 mM Na_2_EDTA, 1 mM EGTA, 1% Triton X-100, 2.5 mM sodium pyrophosphate, 1 mM β-glycerophosphate, 1 mM Na_3_VO_4_, and 1 μg/mL leupeptin). After sonication and centrifugation to remove collagen, samples were reduced with DTT (0.1 M) in 4x loading dye and heated at 70 °C, before being centrifuged at 12,000 rpm for 1 min. Proteins were then separated on a 10% SDS–polyacrylamide gel, using a minigel electrophoresis system (Invitrogen, Waltham, MA, USA), and transferred to a nitrocellulose membrane. The membranes were blocked with 5% nonfat dried milk in TBS-T buffer (10 mM Tris-HCl, pH 8.0, 150 mM NaCl, and 0.3% Tween 20) and incubated overnight at 4 °C with the primary antibodies anti-α-SMA (Cell Signaling Technology, Danvers, MA, USA), and anti-fibronectin and anti-GAPDH (Santa Cruz Biotechnology, Dallas, TX, USA). After three 5 min washes with TBS-T, the membranes were incubated with a secondary antibody for 2 h at room temperature. The membranes were then washed three times with TBS-T for 5 min each, and protein bands were visualized using chemiluminescence substrates (Thermo Fisher Scientific, Waltham, MA, USA) and quantified using NIH ImageJ. Results are expressed as mean ± SEM from three independent experiments, normalized to control.

### 2.4. Immunocytochemistry

RPE cells plated on collagen were first fixed with 4% paraformaldehyde, then washed, permeabilized, and blocked with 5% serum/0.3% Triton X-100/5% BSA in PBS. Next, the samples were incubated with an anti-α-SMA antibody labeled with Alexa Fluor 488 (Thermo Fisher Scientific, Waltham, MA, USA), and phalloidin labeled with CF583 (Biotium, Inc, Fremont, CA, USA) (to stain F actin stress fibers), in 1% BSA-PBS overnight at 4° C. After incubation, the samples were washed three times in 0.1% Triton X-100/PBS. Finally, the samples were mounted with Vectashield (Vector Laboratories, Burlingame, CA, USA) and were imaged under a fluorescence microscope with a digital camera.

### 2.5. Cell Proliferation Assay

Cell proliferation was assessed using a Bromodeoxyuridine (BrdU) cell proliferation assay kit (Cell Signaling Technology; Danvers, MA, USA). Human RPE cells were plated at a density of 5.0 × 10^3^ cells per well in a 96-well plate and cultured in 10% FBS-DMEM for 3 days with *N*-acyl dopamines—PALDA, OLDA, or NADA (3 μM)—or vehicle. After 4 h of BrdU incubation, the cells were fixed, and BrdU incorporation was detected using primary and secondary antibody solutions, followed by wash steps as per the manufacturer’s protocol. BrdU incorporation was measured spectrophotometrically at 450 nm, using a plate reader. Results are presented as mean ± SEM from three independent experiments with different cell batches, with 4 or 5 replicates per condition.

### 2.6. Statistical Analysis

Statistical analyses were performed using GraphPad Prism (version 10) (GraphPad Software, San Diego, CA, USA), with *p*-values < 0.05 considered statistically significant. One-way ANOVA was used to assess differences among treatment groups.

## 3. Results

### 3.1. The Impact of Substitution of Different Fatty Acid Side Chains on the Inhibitory Effects of N-Acyl Dopamines on RPE Cell-Mediated Matrix Contraction

**Figure 2:**
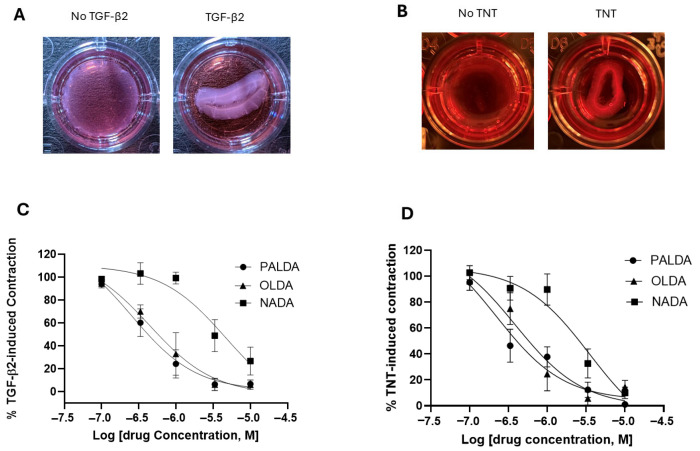
The impact of substitution of different fatty acid side chains on the inhibitory effects of *N*-acyl dopamines on RPE cell-mediated matrix contraction. Cells were treated for 72 h, with either TGF-β2 (10 ng/mL, porcine RPE) or TNT (combined TGF-β2 + TNF-α, each at 10 ng/mL, human RPE), in the presence or absence of *N*-acyl dopamines: *N*-palmitoyl dopamine (PALDA), *N*-oleoyl dopamine (OLDA), or *N*-arachidonoyl dopamine (NADA). After 72 h of treatment, gels were released and allowed to contract for 4 h. (**A**,**B**) are representative images of collagen gels contracted by porcine and human RPE cells, respectively. (**C**,**D**) are the concentration–response curves of *N*-acyl dopamine PALDA, OLDA, and NADA in inhibiting porcine and human RPE cell-mediated collagen matrix contraction, respectively. Data expressed as mean ± SEM.

Building on our previously established porcine RPE cell model and our findings with OLDA [14], we first examined the effect of varying the *N*-acyl side chain structure on the ability of *N*-acyl dopamines to inhibit porcine RPE cell myofibroblast transdifferentiation. Primary cultured porcine RPE cells were plated on a collagen gel and treated with TGF-β2, in the presence or absence of one of three *N*-acyl dopamines: *N*-palmitoyl dopamine (PALDA), *N*-oleoyl dopamine (OLDA), or *N*-arachidonoyl dopamine (NADA) (Figure 1). Upon treatment with TGF-β2 for 72 h, porcine RPE cells potently contracted the collagen gel matrix (Figure 2A). All three *N*-acyl dopamines inhibited the contraction of collagen matrices by porcine RPE cells in a concentration-dependent manner (Figure 2C). The rank of potency of the three *N*-acyl dopamines was PALDA = OLDA > NADA.

To increase clinical relevance and confirm these results in human RPE cells, we adopted a previously published in vitro PVR model, by using human RPE cells treated with both TGFβ2 and TNFα (TNT) to induce myofibroblast transdifferentiation [16]. As shown in Figure 2B, treatment with TNT for 72 h induced a strong collagen matrix contraction, mediated by human RPE cells. In addition, PALDA, OLDA, and NADA, in a concentration-dependent manner, inhibited TNT-induced, RPE cell-mediated collagen matrix contraction (Figure 2D). These *N*-acyl dopamines exhibited a rank of order of potency in human RPE cells that was identical to that in porcine RPE cells, i.e., PALDA = OLDA > NADA.

Overall, these data demonstrated the structure–activity relationship of the fatty acid side chains in the inhibitory effects of *N*-acyl dopamines on RPE cell transdifferentiation.

### 3.2. The Impact of Different Head Groups on the Effects of N-Acyl Amide Derivatives on RPE Cell-Mediated Matrix Contraction

**Figure 3:**
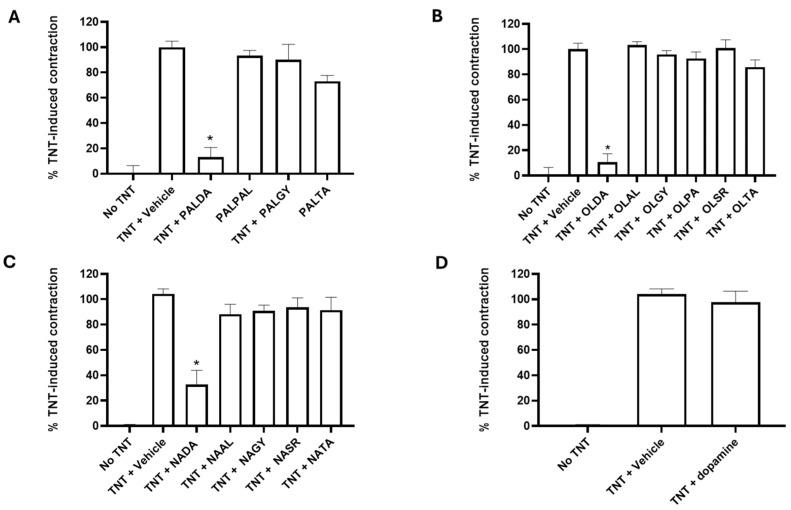
The impact of different head groups on the effects of *N*-acyl amide derivatives on RPE cell-mediated matrix contraction. Primary cultures of human RPE cells were plated on collagen gel matrices and treated with the vehicle or an *N*-acyl amide derivative plus TNT (combined TGF-β2 + TNF-α, each at 10 ng/mL) for 72 h. Gels were released from the plate and allowed to contract for 4 h. (**A**) shows the effects of *N*-palmitoyl amide derivatives: *N*-palmitoyl dopamine (PALDA), *N*-palmitoyl glycine (PALGY), *N*-palmitoyl phenylalanine (PALPA), and *N*-palmitoyl taurine (PALTA). (**B**) shows the effects of *N*-oleoyl amide derivatives: *N*-oleoyl dopamine (OLDA), *N*-oleoyl alanine (OLAL), *N*-oleoyl serine (OLSR), *N*-oleoyl glycine (OLGY), *N*-oleoyl phenylalanine (OLPA), and *N*-oleoyl taurine (OLTA). (**C**) shows the dose–response curve of *N*-arachidonoyl amide derivatives: *N*-arachidonoyl dopamine (NADA), *N*-arachidonoyl L-alanine (NAA), *N*-arachidonoyl glycine (NAG), *N*-arachidonoyl serine (NAS), and *N*-arachidonoyl taurine (NAT). (**D**) shows the effects of dopamine alone, without the acyl side chain. Data are presented as mean ± SEM. * Significantly different from TNT alone, one-way ANOVA with Dunnett’s post-test (*p* < 0.05).

Next, we tested the hypothesis that altering the head group attached to the *N*-acyl side chain could impact upon the ability of these compounds to inhibit RPE cell transdifferentiation. First, we tested palmitic acid side chains conjugated to various head groups. The commercially available compounds and *N*-palmitoyl derivatives *N*-palmitoyl dopamine (PALDA), *N*-palmitoyl phenylalanine (PALPAL), *N*-palmitoyl glycine (PALGY), and *N*-palmitoyl taurine (PALTA) were tested. As shown in Figure 3A, comparing to the treatment with the vehicle, treatment with 3 μM PALDA showed significant inhibition of TNT-induced, RPE cell-mediated contraction of the collagen matrix. In contrast, treatment with non-dopamine head group *N*-palmitoyl derivatives (PALPA, PALGY, or PALTA) showed no difference compared to the vehicle treatment on human RPE cell-mediated collagen matrix contraction.

We next tested the oleic acid side chain derivatives that were conjugated to various head groups. The compounds tested included *N*-oleoyl dopamine (OLDA), *N*-oleoyl alanine (OLAL), *N*-oleoyl glycine (OLGY), *N*-oleoyl serine (OLSR), *N*-oleoyl phenylalanine (OLPA), and *N*-oleoyl taurine (OLTA). As shown in Figure 3B, treatment of human RPE cells with TNT caused contraction of the collagen matrix by the transdifferentiated RPE cells, whereas treatment with 3μM OLDA showed significant inhibition of TNT-induced, RPE-mediated collagen matrix contraction. However, treatment with the non-dopamine head group *N*-oleoyl derivatives (OLAL, OLGY, OLSR, OLPA, and OLTA) showed no significant difference when compared to the vehicle treatment on human RPE cell-mediated contraction of the collagen matrix.

Next, we tested *N*-arachidonoyl side chain derivatives with varied head groups to determine whether substituting dopamine with amino acids would retain the anti-myofibroblast transdifferentiation activity. The compounds tested included *N*-arachidonoyl dopamine (NADA), *N*-arachidonoyl L-alanine (NAAL), *N*-arachidonoyl glycine (NAGY), *N*-arachidonoyl serine (NASR), and *N*-arachidonoyl taurine (NATA). As seen in Figure 3C, none of these *N*-arachidonoyl amide derivatives, except NADA, significantly inhibited human RPE cell-mediated matrix contraction.

The above-described results (Figure 3A–C) clearly demonstrate the importance of the dopamine head group. Therefore, dopamine alone was evaluated to see if the *N*-acyl side chain is necessary for the observed activity of *N*-acyl dopamine. As shown on Figure 3D, dopamine alone was not able to inhibit TNT-induced, human RPE cell-mediated collagen matrix contraction, clearly demonstrating that, while the dopamine head group is required for the inhibitory effect, dopamine alone is not sufficient and the *N*-acyl dopamine structure is required for the inhibitory effect.

### 3.3. The Effects of N-Acyl Dopamines on α-SMA and Fibronectin Expression in Transdifferentiated Human RPE Cells

**Figure 4:**
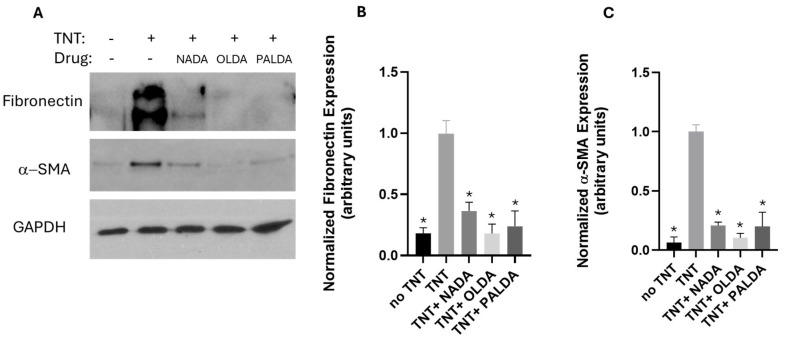
The effects of *N*-acyl dopamines on α-SMA and fibronectin expression in transdifferentiated human RPE cells. Cells were plated on collagen gels and treated with the vehicle or OLDA, PALDA, or NADA, plus TNT (10 ng/mL) for 72 h, released from the plate, and allowed to contract for 4 h. Gels were collected, cells lysed, resolved with SDS-PAGE, immunoblotted, and probed with antibodies against α-SMA, fibronectin, and GAPDH. (**A**) Representative Western blot images showing the expression levels of myofibroblast markers fibronectin and α-SMA and loading control GAPDH. Quantification of band intensity for fibronectin (**B**) and α-SMA (**C**) was performed using NIH Image J, normalized to the loading control GAPDH band intensity, and expressed as fold-change, relative to TNT treatment alone. Data are presented as mean ± SEM. * Significantly different from TNT alone, one-way ANOVA with Dunnett’s post-test (*p* < 0.05).

Western blotting analysis was used to investigate the effect of *N*-acyl dopamines on the expression of myofibroblast cell markers α-SMA and fibronectin. As shown in Figure 4 (original Western blot images are shown in Supplementary Figure S1), following a 72 h TNT treatment, myofibroblast markers α-SMA and fibronectin levels in the human RPE cells had increased significantly. Importantly, treatment with 3 μM of PALDA, OLDA, or NADA significantly decreased TNT-induced α-SMA and fibronectin levels. These data demonstrated the inhibitory effects of the three *N*-acyl dopamines on the myofibroblast markers α-SMA and fibronectin.

### 3.4. The Effects of N-Acyl Dopamines on α-SMA Expression in F-Actin Stress Fibers of Transdifferentiated Human RPE Cells

**Figure 5:**
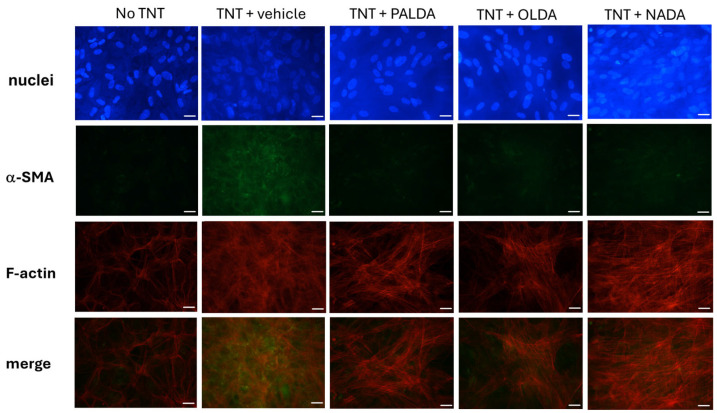
The effects of *N*-acyl dopamines on α-SMA expression in F-actin stress fibers of transdifferentiated human RPE cells. Representative images of immunostaining of F-actin stress fibers (red), α-SMA (green), and their co-expression (orange/yellow in the merged images) are shown. Primary cultured human RPE cells were plated on collagen gels and treated with the vehicle or OLDA, PALDA, or NADA, plus TNT (10 ng/mL) for 72 h. The collagen gels were fixed on the slide and immunostained for F-actin stress fibers with CF583R, labeled phalloidin, and α-SMA with an Alexa Fluor 488, labeled anti-α-SMA, antibodies. The photos were taken using 40× objectives and the scale bars represent 25 micrometers.

As shown in Figure 5, immunofluorescent staining experiments revealed that TNT treatment induced the formation of F-actin stress fibers (red) in RPE cells, and α-SMA (green) is incorporated into these stress fibers (orange/yellow in the merged image). In RPE cells treated with 3 μM of PALDA, OLDA, or NADA, the expression of α-SMA (green) is reduced, and incorporation of α-SMA into F-actin stress fibers (orange/yellow in the merged image) is down-regulated compared to vehicle-treated cells (Figure 5). These data further demonstrated the inhibitory effects of the three *N*-acyl dopamines on the myofibroblast markers.

### 3.5. The Effects of N-Acyl Dopamines on RPE Cell Proliferation

**Figure 6:**
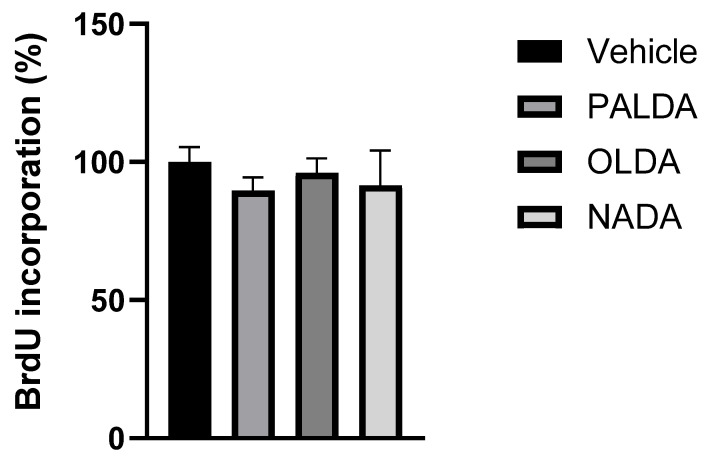
The effects of *N*-acyl dopamines on human RPE cell proliferation. Primary cultured human RPE cells were treated with the vehicle or OLDA, PALDA, or NADA, plus TNT (10 ng/mL) for 72 h. After 4 h of BrdU incubation, cells were fixed, and BrdU incorporation was detected using primary and secondary antibody solutions, followed by wash steps. BrdU incorporation was measured spectrophotometrically at 450 nm, using a plate reader. Data are normalized to vehicle-treated cells and presented as mean ± SEM.

The effect of *N*-acyl dopamines NADA, OLDA, and PALDA on RPE cell proliferation was investigated using the BrdU incorporation assay. As seen in Figure 6, none of these three compounds significantly affected the BrdU uptake by RPE cells as compared to the vehicle, indicating that PALDA, OLDA, and NADA do not affect RPE cell proliferation. These data suggested that rather than affecting cell proliferation, *N*-acyl dopamines are targeting RPE cell transdifferentiation.

## 4. Discussion

With the increasing use of cannabinoids in the medical field and a growing understanding of their benefits, substantial research has been devoted to exploring their potential for treating fibrosis. In this study, we focused on the potential of a new class of endocannabinoid-like compounds, *N*-acyl dopamines, in preventing and treating retinal fibrosis.

RPE cells are considered to be a key source of myofibroblasts, which play an essential role in the development of fibrosis [3,4,5]. Our previous work demonstrated that the *N*-oleoyl dopamine (OLDA), an endocannabinoid-like compound, can inhibit TGF-β2-induced myofibroblast transdifferentiation of porcine RPE cells [14]. The aim of this study was to elucidate the structure–activity relationship among various *N*-acyl amide compounds in their ability to inhibit the myofibroblast transdifferentiation of RPE cells.

In this study, we began our investigation by using primary cultured porcine RPE cells, because porcine eyes exhibit anatomical and functional similarities to human eyes, including comparable retinal organization and RPE characteristics, making them a valuable model for ophthalmic research. Previously, both in vitro and in vivo porcine PVR models have been established in our lab, demonstrating their utility in modeling retinal fibrotic responses [17,18,19]. Porcine PVR models in vivo reproduced key changes observed in human patients, and porcine and human cells in vitro undergo identical fibrotic changes. The data collected using the porcine RPE cells in this study provided us with valuable information regarding the potency of these endocannabinoid-like compounds and will allow us to use this information to further evaluate the therapeutic potential of these compounds, using the porcine PVR models in vivo.

After establishing the efficacy and potency of the three *N*-acyl dopamines in our well-established porcine RPE cell culture model, we transitioned to human RPE cells for validation of clinical relevance and detailed characterization, including using two molecular markers of myofibroblast transdifferentiation. Most of our experiments were conducted with a modification of a previously published in vitro human PVR model, using primary cultures of human RPE cells and stimulated with two cytokines, TGF-β2 and TNF-α [16]. It is worth mentioning that the adult human primary RPE cells used for this study were isolated, cultured, and validated by the Eye Bank of New York, using the originally published procedures for these cells [16].

In the following sections, we will discuss the structure–activity relationship, functional significance, and potential molecular targets of *N*-acyl dopamines as inhibitors of myofibroblast transdifferentiation of RPE cells. We will also discuss the novelty and limitations of this study and future directions.

### 4.1. Structure–Activity Relationship of N-Acyl Amide Derivatives on Inhibiting Myofibroblast Transdifferentiation of RPE Cells

Using primary cultures of both porcine and human RPE cells, we first examined the influence of the length and degree of saturation of the *N*-acyl tail attached to the dopamine head group on the ability to inhibit RPE cell myofibroblast transdifferentiation. PALDA has a saturated fatty acid side chain (16 carbons), OLDA is monounsaturated (18 carbons), and NADA is polyunsaturated (20 carbon side chain with four double bonds). Among these tested compounds, PALDA and OLDA were equipotent in inhibiting the TNT-induced contraction of RPE cells, and NADA was less potent than PLDA and OLDA. This data suggests that a shorter length and more saturated fatty acid side chain favors greater potency of these *N*-acyl dopamine compounds.

We then examined the effect of substituting dopamine with varied head groups, such as alanine, serine, glycine, phenylalanine, and taurine, conjugated with an amide bond to palmitoyl, oleoyl, and arachidonoyl fatty acid side chains. Our results showed that replacing the dopamine moiety of an *N*-acyl amide derivative with any of the above tested amino acids led to a complete loss of their ability to inhibit TNT-induced, RPE cell-mediated matrix contraction. These data reinforce the notion that the dopamine head group is crucial for these ligands to exert their effects on blocking the myofibroblast transdifferentiation of RPE cells. However, dopamine alone did not have any activity in RPE cell transdifferentiation, demonstrating that the fatty acid chains are necessary as well.

Overall, our data demonstrate that both the dopamine head group and the acyl side chain are important for the *N*-acyl dopamines in inhibiting the myofibroblast transdifferentiation of RPE cells. It seems that the dopamine head group is absolutely required, whereas the length and degree of saturation of the fatty acid side chains determine the potency of these *N*-acyl dopamine compounds.

Furthermore, in cell proliferation assays, none of the *N*-acyl dopamines tested had any significant effects on RPE cell proliferation, as indicated by BrdU incorporation. These data suggest that the ability of these compounds to inhibit RPE cell contraction is likely not due to their effects on cell growth but, rather, attributed to the specific inhibition of myofibroblast transdifferentiation.

### 4.2. Functional Significance and Potential Molecular Targets of N-Acyl Dopamines as Inhibitors of Myofibroblast Transdifferentiation of RPE Cells

In this study, we discovered that several endocannabinoid-like compounds, *N*-acyl dopamines, are effective in inhibiting myofibroblast transdifferentiation of RPE cells. This discovery holds significant therapeutic potential for fibrotic retinal conditions, such as PVR, a disease currently lacking approved medications for treatment and prevention, especially since myofibroblast transdifferentiation of RPE cells is considered to play an essential role in the fibrotic process [4,16]. Multiple steps of the PVR process can be targeted for adjunctive treatment [20]. For example, methotrexate, which targets cell proliferation and inflammation, is promising as an adjunct treatment for PVR in clinical trials. Since *N*-acyl dopamines primarily target the phenotypic change to myofibroblasts, it may offer an alternative novel pharmaceutical approach for preventing and treating PVR.

The lack of approved treatment for PVR prevention thus far is likely, at least in part, due to drugs not maintaining sufficient therapeutic doses, either by a limited number of drug applications and/or short retention time of applied drugs within the vitreous cavity. We have previously demonstrated this using a different drug, dasatinib, in which an injection of bulk solution failed, but a sustained delivery system significantly reduced the incidence of PVR [17]. We believe a sustained delivery system of *N*-acyl dopamine that can overcome the potential issues of a fast clearance rate (due to a small molecular weight) can potentially have a significant impact on the prevention of primary and recurring PVR by blocking the conversion of RPE cells to myofibroblasts.

Furthermore, the formation of fibrotic tissues through transdifferentiation of resident cells into myofibroblasts can happen across many organ systems in response to injury. Thus, inhibiting this process can be an effective therapeutic approach for preventing excessive scarring and tissue remodeling in other organ systems. Our findings suggest that *N*-acyl dopamines may suppress this process in RPE cells. If this effect can be translated to other cell types, *N*-acyl dopamines could help to mitigate pathological wound healing and fibrotic progression, not only in the eye, but also expanding into the therapeutic landscape for other fibrotic diseases.

There are several potential molecular targets that *N*-acyl dopamines may act upon to produce their inhibitory effects on the transdifferentiation of RPE cells. These possible molecular targets include the CB1 cannabinoid receptor [12,21,22], TRPV1 [12,23,24], and GPR6 [11].

The CB1 cannabinoid receptor is a well-known member of the endocannabinoid system. Previously, ligand binding studies conducted using rat brain membranes have found that among the three acyl-dopamines we tested, NADA had the highest affinity for CB1, OLDA exhibited a moderate binding affinity, and PALDA had low affinity [21]. In terms of CB1 agonist activity, it has been reported that NADA is a CB1 receptor agonist, but it is unknown if OLDA and PALDA act as agonists or antagonists [12,25]. The involvement of CB1 in fibrotic diseases is well-established [26,27]. In addition, it has been shown that CB1 is expressed in RPE cells [28,29]. Therefore, it is possible that *N*-acyl dopamines tested in this study produce their effects on RPE cell transdifferentiation through CB1. However, there is also evidence indicating that CB1 may not be a target for these compounds to exhibit their anti-fibrotic effects. First, the rank order of potency we observed for these compounds for inhibiting myofibroblast transdifferentiation (PALDA = OLDA > NADA) does not match their CB1 affinity (NADA > OLDA > PALDA). Also, it is well-established that CB1 agonists are profibrotic and CB1 antagonists are antifibrotic [26,27], but NADA exhibited antifibrotic properties in this study, despite being a CB1 agonist.

The TRPV1 receptor is a type of transient receptor potential channel and is a non-selective cation channel that allows for the passage of cations, with particularly high permeability for Ca^2+^ [30,31]. Previous research has demonstrated that OLDA and NADA are TRPV1 agonists, whereas PALDA does not directly activate TRPV1 [12,21,23,24]. However, PALDA exhibited “entourage” effects by enhancing the activity of TRPV1 agonists [23]. Recent studies have suggested that TRPV1 is a therapeutic target for fibrotic diseases in a number of organ systems [32]. TRPV1 is also known to be expressed in RPE cells, where it plays an important functional role [33]. Therefore, TRPV1 might mediate the inhibitory effects of these *N*-acyl dopamines, to inhibit myofibroblast transdifferentiation of RPE cells. On the other hand, PALDA exhibited equal potency as OLDA in the current study, whereas PALDA does not activate TRPV1. This seems to be at odds with the notion that TRPV1 is a molecular target for PALDA in RPE cells.

Our previous research has shown that *N*-acyl dopamines, including PALDA, OLDA, and NADA, act as inverse agonists for GPR6, an orphan G protein coupled receptor that is phylogenetically related to CB1 and CB2 cannabinoid receptors [11]. In the current study, these three *N*-acyl dopamines exhibited structure–function relationships that were very similar to those shown on GPR6; i.e., the dopamine head group is crucial for their activity, whereas the fatty acid side chain length and degree of saturation determine their potency relative to each other. Also, the rank order of potency of these compounds observed in the current study matches those for GPR6 activation assays that we reported previously [11]. These data point toward the possibility that GPR6 could be involved in the inhibitory effects of *N*-acyl dopamines on myofibroblast transdifferentiation. However, whether GPR6 is expressed in the RPE cells, and its role in the actions of *N*-acyl dopamines, remain to be determined.

### 4.3. Novelty and Limitations of the Study and Future Directions

Overall, this is the first systematic structure–activity relationship study of *N*-acyl dopamines in RPE fibrosis. Our data indicated the potential of these compounds as a novel class of agents targeting myofibrobast transdifferentiation of RPE cells. The limitations of this study include the lack of in vivo data and detailed mechanisms of actions. Also, we did not perform long-term toxicity studies, even though we found that these compounds do not affect RPE cell proliferation. Future studies using animal models of PVR are warranted to validate therapeutic efficacy, and to ensure these compounds are safe to use. In addition, more mechanistic studies are needed, to elucidate detailed molecular signaling cascades for *N*-acyl dopamines to inhibit RPE cell transdifferentiation.

## 5. Conclusions

In summary, we identified PALDA, OLDA, and NADA, three structurally related *N*-acyl dopamine derivatives with varied fatty acid side chains that had similar inhibitory effects on RPE cell transdifferentiation. The rank order of potency for these *N*-acyl dopamines depends on the length and degree of saturation of their fatty acid side chains. Importantly, *N*-acyl amide derivatives lack the dopamine head group failed to reproduce this effect, suggesting the dopamine moiety is critical for the observed inhibitory effects on myofibroblast transdifferentiation of RPE cells. The data from this study point to the therapeutic potential of these endocannabinoid-like compounds in preventing and treating fibrotic retinal conditions such as PVR. Further studies are warranted to elucidate the detailed molecular mechanisms by which these *N*-acyl dopamines exert their actions, and if these endocannabinoid-like compounds inhibit pathogenic retinal fibrosis in vivo.

## Data Availability

Data are contained within the article.

## Supplementary Materials

The following supporting information can be downloaded at: https://www.mdpi.com/article/10.3390/biom15111526/s1, Figure S1: The original western blot images of Figure 4.

## Abbreviations

The following abbreviations are used in this manuscript:

RPE	Retinal pigment epithelial
PVR	Proliferative vitreoretinopathy
PALDA	*N*-palmitoyl dopamine
OLDA	*N*-oleoyl dopamine
NADA	*N*-arachidonoyl dopamine
