# Supplementary figures and images for: Structure–Activity Relationships of N-Acyl Dopamines in Inhibiting Myofibroblast Transdifferentiation of Retinal Pigment Epithelial Cells
Source: Biomolecules. 2025 Oct 30;15(11):1526. doi: 10.3390/biom15111526 (PMC12650079; doi:10.3390/biom15111526)

Fibronectin

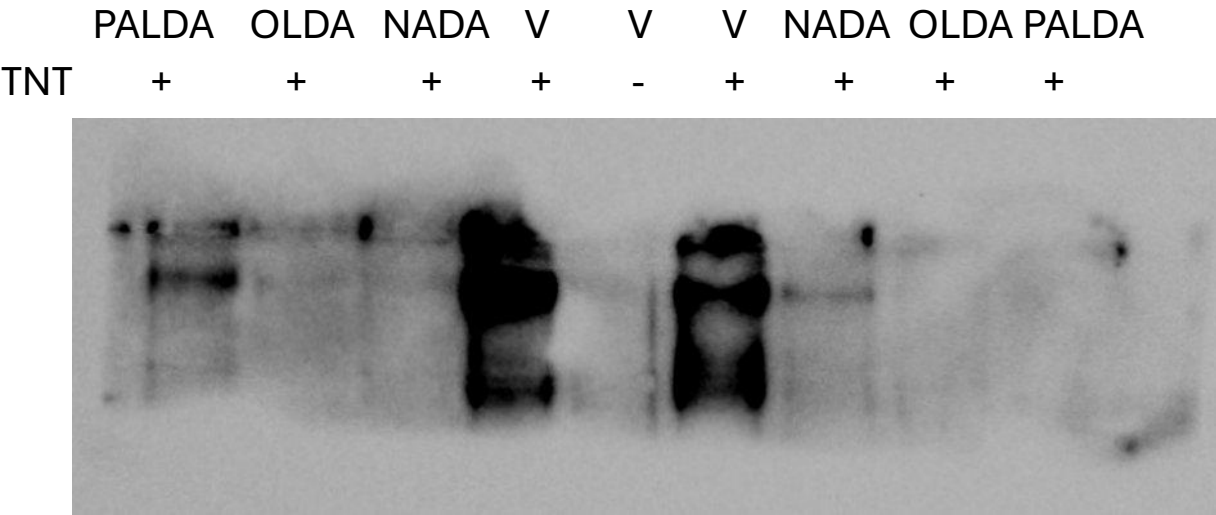

GAPDH

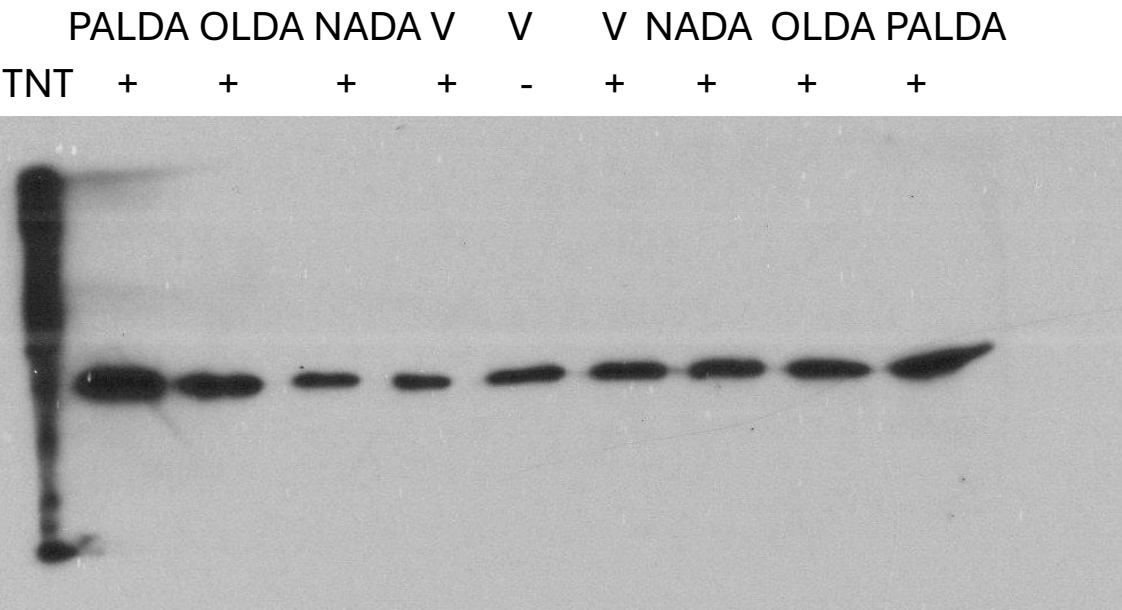

$\alpha$ SMA

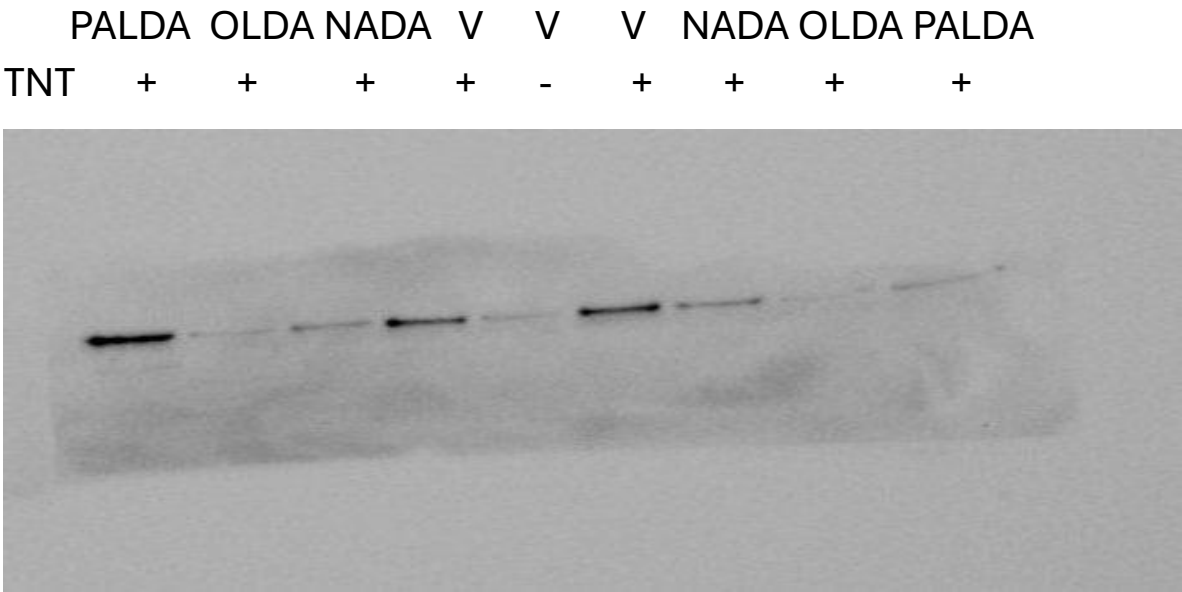

Figure S1:  
The original western blot images of Figure 4

Supplement: Supplementary file 1 [file biomolecules-15-01526-s001.zip › biomolecules-3906868-supplementary.pdf]
